# The healing of alveolar bone defects with novel bio-implants composed of Ad-BMP9-transfected rDFCs and CHA scaffolds

**DOI:** 10.1038/s41598-017-06548-7

**Published:** 2017-07-25

**Authors:** Li Nie, Xia Yang, Liang Duan, Enyi Huang, Zhou Pengfei, Wenping Luo, Yan Zhang, Xingqi Zeng, Ye Qiu, Ting Cai, Conghua Li

**Affiliations:** 1 Stomatological Hospital of Chongqing Medical University, Chongqing, 401147 China; 2 Chongqing Key Laboratory of Oral Diseases and Biomedical Sciences, Chongqing Municipal Key, Chongqing, 401147 China; 3 Laboratory of Oral Biomedical Engineering of Higher Education, Chongqing, 401147 China; 4 Department of Laboratory Medicine, the Second Affiliated Hospital of Chongqing Medical University, Chongqing, 400010 China

## Abstract

Cells, scaffolds, and growth factors play important roles in bone regeneration. Bone morphogenetic protein 9 (BMP9), a member of BMP family, could facilitate osteogenesis by regulating growth factors and promoting angiogenesis. Similar to other stem cells, rat dental follicle stem cells (rDFCs), the precursor cells of cementoblasts, osteoblasts and periodontal ligament cells, can self-renew and exhibit multipotential capacity. Coralline hydroxyapatite (CHA) has good biocompatibility and conductivity required for bone tissue engineering. Here, we reported that BMP9 could enhance the osteogenic differentiation of rDFCs in cell culture. Moreover, our results suggested that BMP9 acted through the Smad1/5/8 signaling pathway. We also produced a novel scaffold that encompasses bio-degradable CHA seeded with recombinant adenoviruses expressing BMP9-transfected rDFCs (Ad-BMP9-transfected rDFCs). With this implant, we achieved more alveolar bone regeneration in the alveolar bone defect compared to blank group, CHA group and rDFCs group. Our results provided a novel bio-implants composed of Ad-BMP9-transfected rDFCs and CHA scaffolds and its mechanism is regarding the activation of Smad1/5/8 signaling pathway in BMP9-induced rDFCs osteogenesis.

## Introduction

Periodontitis is one of the most common oral diseases^1^. It has been characterized as a chronic infectious and inflammatory disease, which affects periodontal tissues and eventually leads to tooth loss^2^. The ultimate goal of all periodontal treatment is to reconstruct the periodontal tissue and restore its functions^3^. However, the current conventional methods (i.e. supra-and sub-gingival scaling, root planning, and surgical treatment) could treat periodontitis but have shown limited effectiveness in regenerating periodontal tissues regeneration^4–6^. Recently, tissue engineering techniques have made enormous progress in the field of bone regeneration through utilizing a combination of three-dimensional (3D) scaffolds, cells and growth factors^7–9^.

In 2008, Yao *et al*. proved that dental follicle stem cells (DFCs), derived from dental follicle, could contribute to the development of periodontal tissue^10^. Similar to other stem cells, the DFCs display both self-renewal and multipotential differentiation capacity^11^. It was reported that DFCs could express the markers of mesenchymal stem cells (MSCs) including Nestin, notch-1 and Stro-1^12, 13^. Further studies have shown that DFCs could differentiate into osteoblasts, cementoblasts, neuroblasts, fibroblasts and lipoblasts^11, 14, 15^.

Compared to other family members, BMP9 had more potent in inducing osteogenic differentiation of stem cells^16–19^. Xu^20^ confirmed that BMP9 induced osteogenic differentiation in mesenchymal stem cell line C3H10T1/2. Randall^21^ showed that BMP9 could induce large volumes of ectopic bone formation adjacent to spine *in vivo*. The question still remains on whether BMP9 could promote periodontal alveolar bone regeneration. Our previous study demonstrated that BMP9 could stimulate the expression of osteogenic differentiation genes in rDFCs. However, further animal studies are intensely necessary to further characterize the osteogenetic potential of BMP9-induced cells *in vivo*. In previous researches, Wei Shui^22^ reported that the application of BMP9-transduced C2C12 cells and type I collagen sponge or HA-TCP scaffold achieved robust and mature cancellous bone formation. Jing Wang^23^ indicated that the mineralization and maturity of BMP9-induced ectopic bone formation could be enhanced by Nell1 expression. Ye^24^ found that thermoresponsive polydiolcitrate-gelatin scaffold facilitated BMP9-induced osteogenic differentiation of MSCs *in vivo* and could promote the formation of well-ossified and vascularized trabecular bone-like structures in a mouse model of ectopic bone formation. Viral vectors for gene delivery are the most popular vectors used in research due to their high efficiency^17^. Delivery of the gene by adenoviral vectors can be better controlled and more efficient than delivery of the protein^17^. Recombinant human bone morphogenetic protein 9 (rhBMP9) exhibited significantly weaker osteogenic activity, compared with that induced by recombinant adenoviruses expressing BMP9 (Ad-BMP9)^19^. For these reasons, we considered investigating the possibility of seeding BMP9-induced rDFCs on scaffolds to repair periodontal alveolar bone defect *in vivo*.

Members of the BMP family could induce osteogenic differentiation of MSCs through Smad-dependent^25, 26^ or Smad-independent signaling pathway^27, 28^. Researchers have demonstrated Smad-dependent signal pathway begins with binding the BMP type I receptors to the BMP type II receptors, then the activated complex receptors promotes the phosphorylation of Smad1/5/8. The phosphorylated Smad1/5/8 forms a complex with Smad4 in the nucleus, which activates the transcription of target osteogenic genes^29^. And, we have demonstrated that BMP9 could effectively promote the osteogenic differentiation of rDFCs by regulating P38 and ERK1/2 MAPK manner, a Smad-independent signaling pathway^11^. However, it still remains unclear whether BMP9 could induce the osteogenic differentiation in rDFCs by activating the Smad-dependent signaling pathway directly or indirectly.

Coralline hydroxyapatite (CHA) scaffolds which have good biocompatibility and conductivity, have been widely applied in tissue engineering^30–32^. CHA scaffolds applied in clinical studies are manufactured by the hydrothermic conversion of hydroxyapatite to calcium carbonate exoskeletons of sea coral^30–32^. In recent studies, CHA scaffolds were used as porous materials to promote bone regeneration^33^. Interestingly, it was shown that human MSCs could adhere to the surfaces of CHA, then proliferate and differentiate^34^. For this reason, we considered investigating the possibility of seeding rDFCs on CHA scaffolds.

To date, there is no study that investigates the role of rDFCs transfected with Ad-BMP9 and co-cultured with CHA in periodontal alveolar bone regeneration and the osteogenic mechanism of BMP9-induced rDFCs. In this study, we transfected rDFCs with Ad-BMP9 then culture those cells on CHA scaffolds. The aims of this study were as follows: (i) to assess whether Ad-BMP9 or CHA can induce osteogenesis in rDFCs, (ii) to determine whether the combination of Ad-BMP9-transfected rDFCs and CHA scaffolds can effectively promote alveolar bone formation *in vivo* and (iii) to identify whether Smad1/5/8 signaling pathway is involved in the process of BMP9-induced osteogenesis in rDFCs.

## Result

### The primary rDFCs and rDFCs transfected with Ad-BMP9

**Figure 1:**
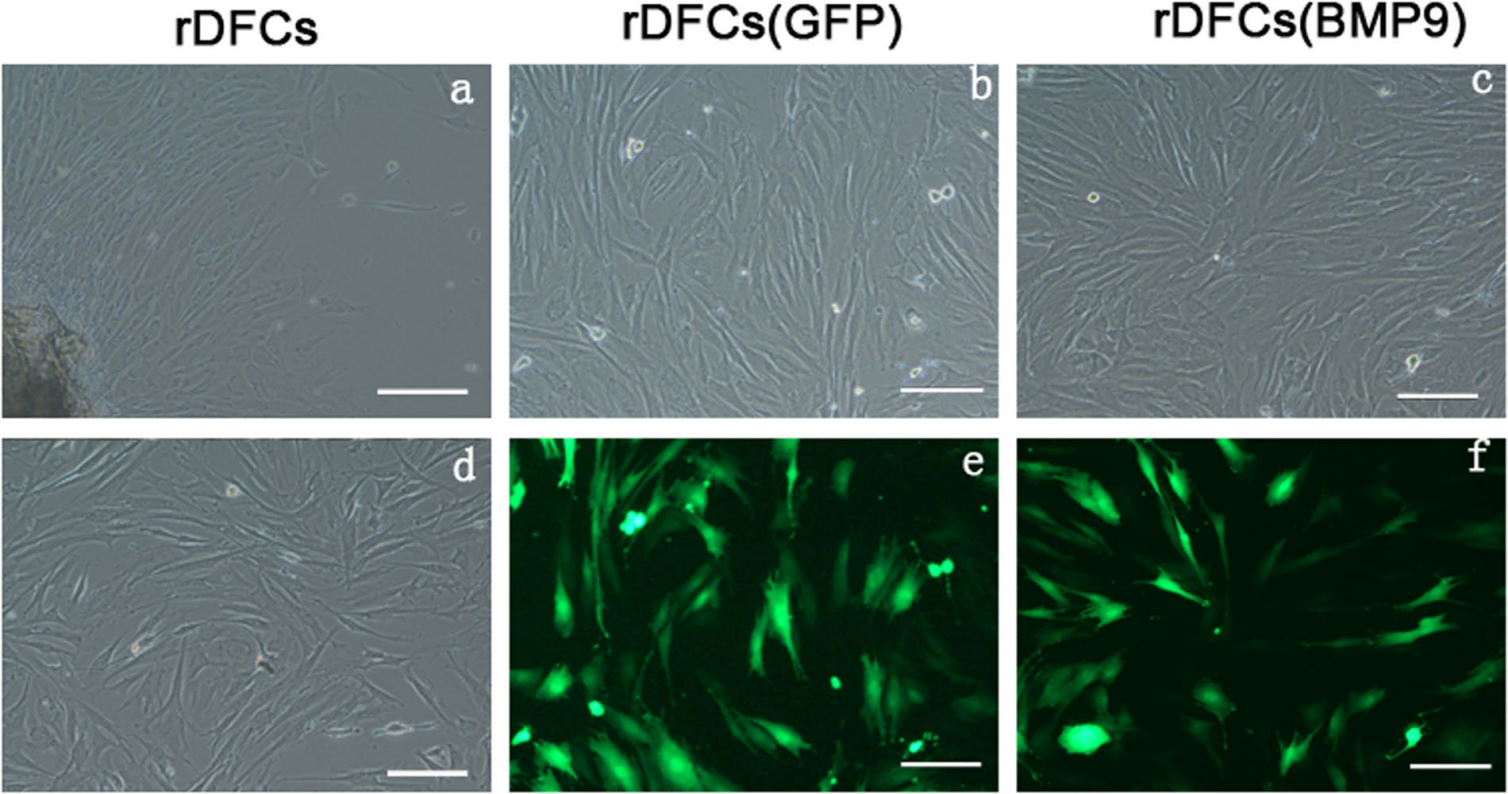
The primary rDFCs and rDFCs transfected by Ad-BMP9. The primary and the 3^rd^ passage rDFCs showed in Fig. 1a and d. The shape and fluorescence of rDFCs transfected with Ad-BMP9 or Ad-GFP was also showed in Fig. 1b,e,c and f. Blank bars: 50 μm.

As shown in Fig. 1a–c, the primary rDFCs were polygonal and fusiform. After the third passage, rDFCs appeared fibroblast-like fusiform (Fig. 1d). 1 to 3 cell nucleoli and highly-densed granules can be observed in the cytoplasm (Fig. 1d). Green fluorescence could be detected in rDFCs 24 hours after adenovirus expressing bone morphogenetic protein 9 (Ad-BMP9) or adenovirus green fluorescence (Ad-GFP) transfection. After 48 hours in culture, the expression of fluorescence increased and the transfection efficiency was about 70%-80% without changing in cells shape (Fig. 1e and f). The green fluorescence was regarded as a mark of Ad-BMP9-transfected rDFCs successfully.

### BMP9-induced osteogenic differentiation of rDFCs

**Figure 2:**
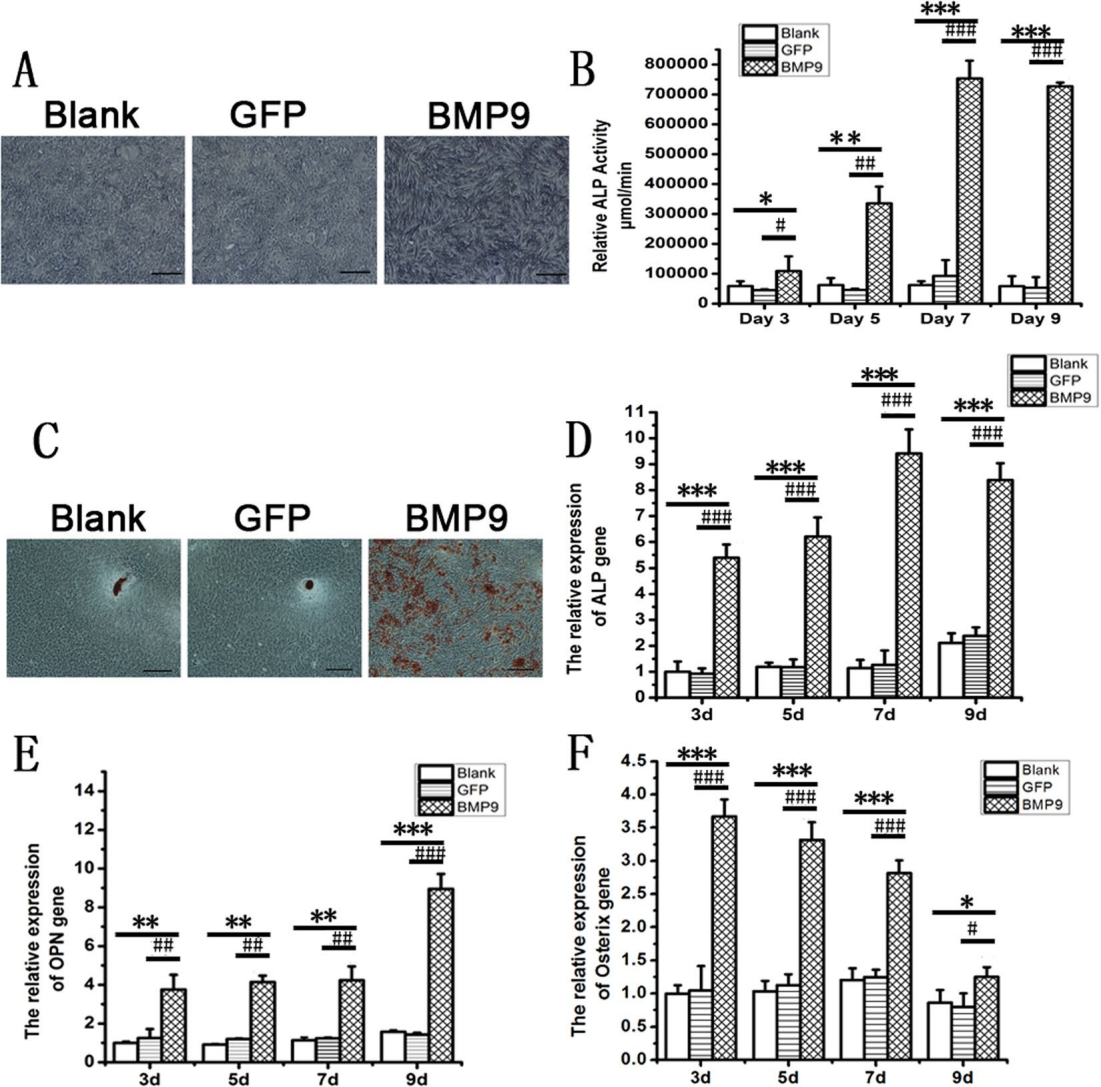
BMP9-induced osteogenic differentiation of rDFCs. (**A**) ALP staining intensity was enhanced in BMP9 group when compared with the blank group and GFP group. Bars: 50 μm. (**B**) ALP activity was also significantly increased on days 3, 5, 7, 9 in BMP9 group when compared with the blank group and GFP group. (**C**) Calcium deposition was stained by Alizarin red staining. More calcified nodules were found in BMP9 group when compared with the blank group and GFP group. Bars: 50 μm. (**D**,**E** and **F**) the relative expression of ALP, OPN, and Osterix were detected by Real Time-*q*PCR. *P < 0.05, **P < 0.01, ***P < 0.001, compared with blank group, ^#^P < 0.05, ^##^P < 0.01, ^###^P < 0.001, compared with GFP group.

To assess the osteogenic effect of BMP9, we performed alkaline phosphatase staining (ALP staining) experiments in the cultured rDFCs. The elevated ALP activity could be distinguished from day 5 in BMP9 group and reached the peak level at day 7 (Fig. 2A,B). We further quantified the mRNA level of alkaline phosphatase (ALP), an important early osteogenic marker^11, 35^, by means of Real Time-*q*PCR. As expected, the expression showed the similar pattern with the maximal expression on day 7 (Fig. 2B). Osterix, one of the main osteogenic determinants^36^, remained at a high level on day 3 and gradually decreased from day 3 to day 9. The expression of osteopontin (OPN), the late osteogenesis marker^11, 35, 37^, continued to increase from the third day and reached the maximal level on day 9. Alizarin red staining was performed to detect the calcium deposition. Alizarin Red staining showed some large and red calcified nodules in BMP9 group on day 14. In blank and GFP groups, the calcified nodules were occasionally found and lightly stained (Fig. 2C). The results suggested that BMP9 could promote the osteogenesis of rDFCs.

### Osteogenic differentiation of rDFCs promoted by CHA

**Figure 3:**
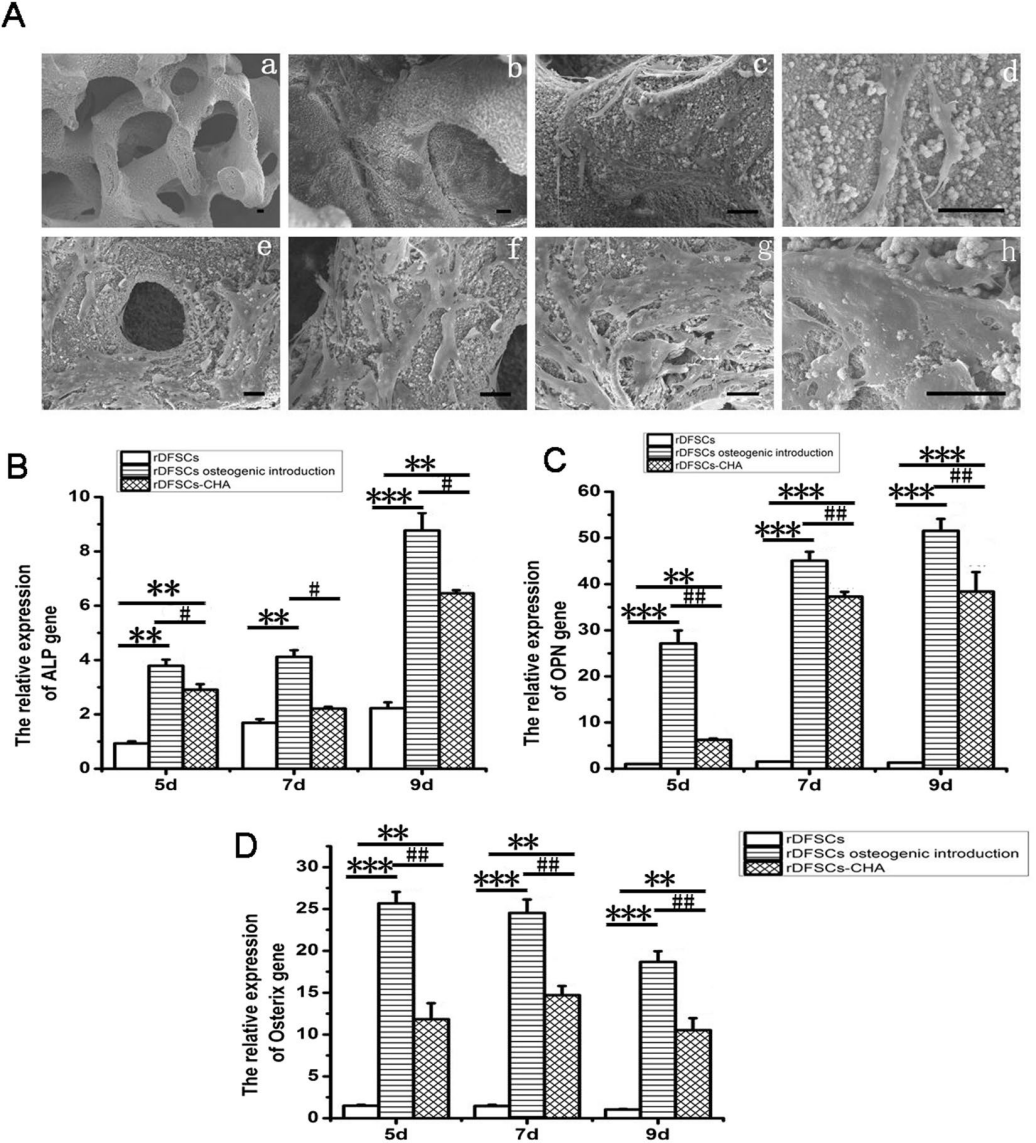
CHA promoted osteogenesis of rDFCs. (**A**) The morphology of CHA and cells on or in CHA was observed by SEM. The construction of CHA scaffold was porous and the pores were connected. rDFCs could adhere to CHA, then proliferate and differentiate on the surface and side wall of visual pores of CHA scaffolds. Cell shape appeared viable when being co-cultured for 24 hours (a, b, c and d). The accumulation of cells and secretory extracellular matrix began to contact with each other and adhered to the surface of CHA on day 4 (e and f). Extracellular matrix fully covered the surface of CHA scaffolds on day 7 (g and h). Bars: 30 μm. (**B**,**C** and **D**) Real Time-*q*PCR clarified that CHA scaffolds altered rDFCs osteogenic gene expression on 5^th^, 7^th^, 9^th^ days. **P < 0.01, ***P < 0.001, compared with rDFCs group, ^#^P < 0.05, ^##^P < 0.01, compared with osteogenic induction group.

The surface structure of CHA scaffolds was illustrated by scanning electron micrographs (SEM). The results revealed that CHA scaffolds had lots of pores with a diameter ranging from 100 to 600 μm (Fig. 3A). Furthermore, SEM also showed that the cells adhered to the surface and side wall of visual pores of CHA scaffolds (Fig. 3A). First sign of adhesion of rDFCs to the CHA scaffolds appeared after 24 hours in co-culture (Fig. 3A,b,c and d). All the cells showed sufficient adhesion with polygonal and fusiform shape. Moreover, pseudopodium of cells could be observed (Fig. 3A,c and d).The number of cells on the surface and the side walls of visual pores gradually increased over time. The accumulation of cells and secretory extracellular matrix began to contact with each other and adhered to the surface of CHA on day 4 (Fig. 3A,e and f). Extracellular matrix fully covered the surface of CHA scaffolds on day 7 (Fig. 3A,g and h).

To assess the effect of CHA on promoting osteogenic differentiation potential of rDFCs, we distributed into 3 groups by culture them in normal growth medium (rDFCs group), osteogenic differentiation medium (osteogenic induction group), or co-culture with CHA scaffolds in normal growth medium (CHA group). After 3, 5, 7 days, we extracted the cells from culture for gene expression study (Fig. 3B,C and D). The expression of ALP, a well-established early osteogenic marker^11, 35^, elevated dramatically in CHA group compared to the control group on day 5 and 7 (P < 0.01). But, it was still not comparable to the ALP expression level in the osteogenic induction group (P < 0.1). Cells cultured with CHA scaffolds exhibited higher level expression of late osteogenic gene, OPN^11, 35, 37^, compared with those cells without any treatment (rDFCs group) on day 7 and 9 (P < 0.001), though lower level than cells stimulated by osteogenic differentiation medium (osteogenic induction group)on day 5, 7 and 9 (P < 0.01) (Fig. 3C). The expression levels of Osterix, one of the main osteogenic determinants^36^ were significantly higher in CHA group than those in the rDFCs group on day 5, 7 and 9 (P < 0.01), and lower than those in osteogenic induction group on day 5, 7 and 9 (P < 0.01) (Fig. 3D). These results suggest that CHA scaffolds enhance the osteogenic differentiation of rDFCs, but it is not as effective as the osteogenic induction medium.

### New bone formation promoted by CHA and rDFCs transfected with Ad-BMP9 *in vivo*

**Figure 4:**
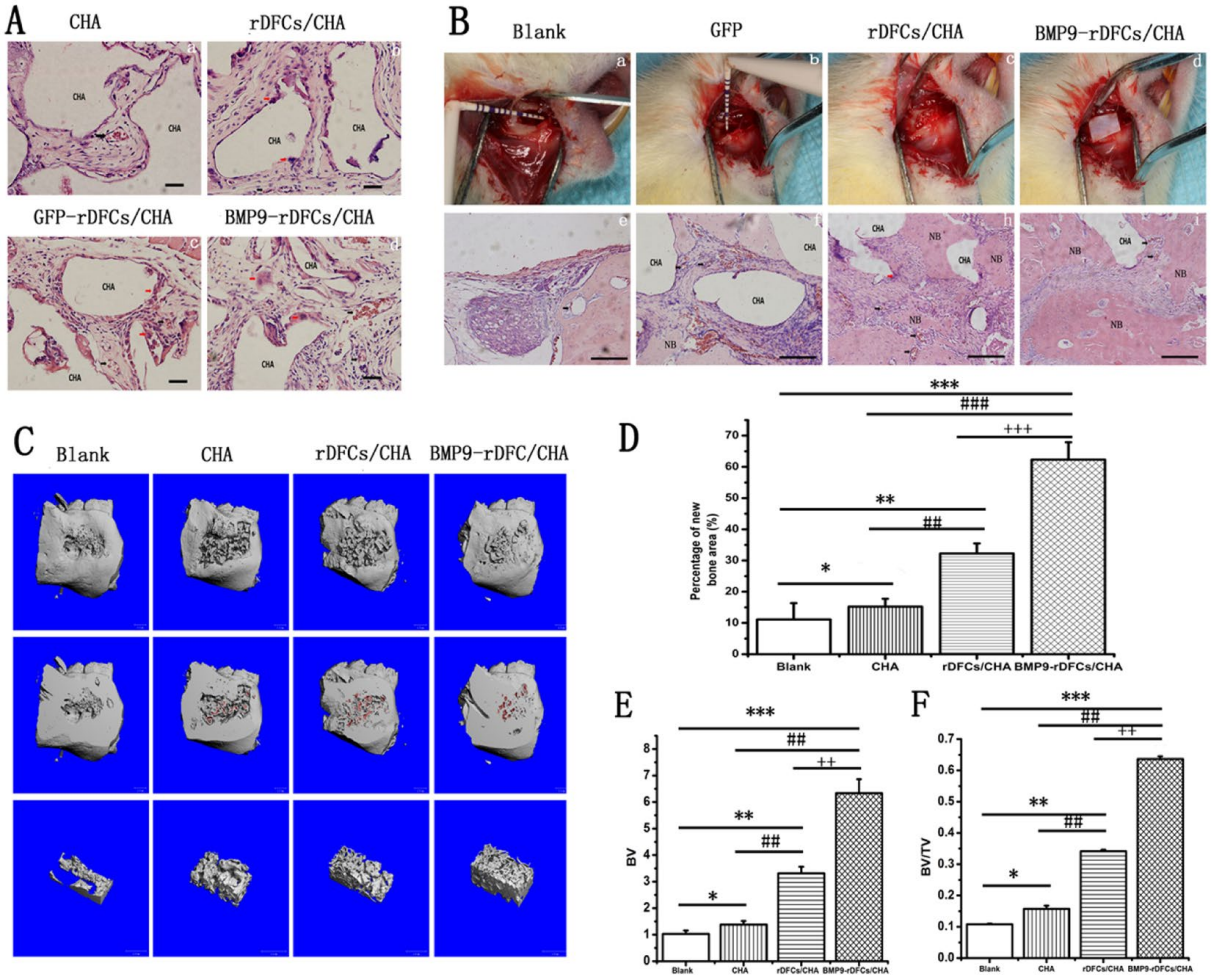
New bone formations *in vivo*. (**A**) Hematoxylin and eosin staining for sections of ectopic implantation. (**B**) The surgical procedure of implantation in alveolar bone defects of rats was showed in Fig. 4a,b,c,d. Hematoxylin and eosin staining for sections of new bone formation in alveolar bone defect in the down-panel of Fig. 4e,f,g,h. Blank bars: 5 μm. CHA: CHA scaffold materials. NB: new bone formation. Black arrow: blood vessel. Red arrow: macrophage. (**C**) The evaluation of new alveolar bone by Micro CT and 3D reconstruction. The morphology of new bone formation was revealed by 3D reconstructed images in blank group, CHA group, rDFCs/CHA group and BMP9-rDFCs/CHA group. CHA scaffolds were in red. (**D**) Percentage of new bone area. (**E**) BV: new bone volume. (**F**) BV/TV: new bone ratio of volume of new formation bone and volume of VOI. *P < 0.05, **P < 0.01, ***P < 0.001 compared to blank group, ^#^P < 0.05, ^##^P < 0.01, ^###^P < 0.001 compared with CHA group, ^++^P < 0.01, ^+++^P < 0.001 compared with rDFCs/CHA group.

In order to evaluate the osteogenic potential of BMP9-treated rDFCs *in vivo*, we prepared CHA, rDCFs/CHA, GFP-rDCFs/CHA, and BMP9-rDCFs/CHA implants for animal experiments. First, we chose to use an ectopic bone formation animal model, as this model would allow us to test whether the novel compound of Ad-BMP9-transfected rDFCs-CHA could supports bone formation in such an environment without any stimulation. And we performed ectopic implantation under the skin of immunodeficient mice. After 6 weeks, the implants were removed and decalcified. Paraffin sections were collected and stained with hematoxylin and eosin for observation. However, there is no evidence of mature new bone formation observed around the CHA scaffolds in all the four groups (Fig. 4A). Interestingly, we found fibrous tissues and immature fibrous-like bone with macrophagocytes and plenty of blood vessels in rDFCs/CHA group and BMP9-rDFCs/CHA group. And there were only fibrous tissues in CHA group. The alveolar bone defect was treated with or without those implants in a rat model in order to test whether the compound implantation would support bone formation to restore alveolar bone defect in an acute traumatic inflammatory environment. After 6 weeks, HE staining was done. A little bone formation was observed in blank and CHA group. The defect filled with fibrous connective tissues showed only a little new alveolar bone along the edge of host bone in blank group (Fig. 4B,e). In CHA group, defects filled with CHA showed a little bone formation with fibrous connective tissue around scaffolds (Fig. 4B,f). Meanwhile, amount of new bone formation and irregularly arranged fibrous connective tissues were also found in rDFCs/CHA treated animals (Fig. 4B,h). Surprisingly, there were numerous new bone formations surrounding the CHA scaffolds and plenty of blood vessels in the defect area with BMP9-rDFCs/CHA implants as showed (Fig. 4B,i). The findings were accordance with the result of image analysis of new bone percentage (Fig. 4E). There were significant differences between BMP9-rDFCs/CHA group and all the other groups (P < 0.01). There was more new bone in rDFCs/CHA group than that in CHA group (P < 0.01) and blank group (P < 0.01). And the result showed less new bone in blank group compared to CHA group (P < 0.05). Taken together, the novel compound implant (BMP9-rDFCs/CHA) achieved the best effects in the restoration of alveolar bone defect.

The 3D reconstruction image from Micro CT data was also used to analyze the new bone formation in each group. A bone defect was still visible in blank group. Meanwhile, the defects treated with CHA, rDFCs/CHA or BMP9-rDFCs/CHA implants were filled with newly generated bones and CHA scaffolds. The results: bone volume (BV) and bone ratio (volume of new formation bone /volume of interest, BV/TV) also confirmed that there were more volume of new bones in the BMP9-rDFCs/CHA implant group compared to rDFCs/CHA group (P < 0.01), CHA group (P < 0.01) and blank group (P < 0.001) (Fig. 4D and E). New bone could also be found in rDFCs/CHA, and was obviously more than in CHA group and blank group compared to CHA group and blank group (P < 0.01) (Fig. 4D and E). Those results suggested that BMP9-rDFCs/CHA implantation could significantly promote alveolar bone regeneration.

### Regulation of osteogenic differentiation of Ad-BMP9-transfected rDFCs by Smad1/5/8 signaling pathway

**Figure 5:**
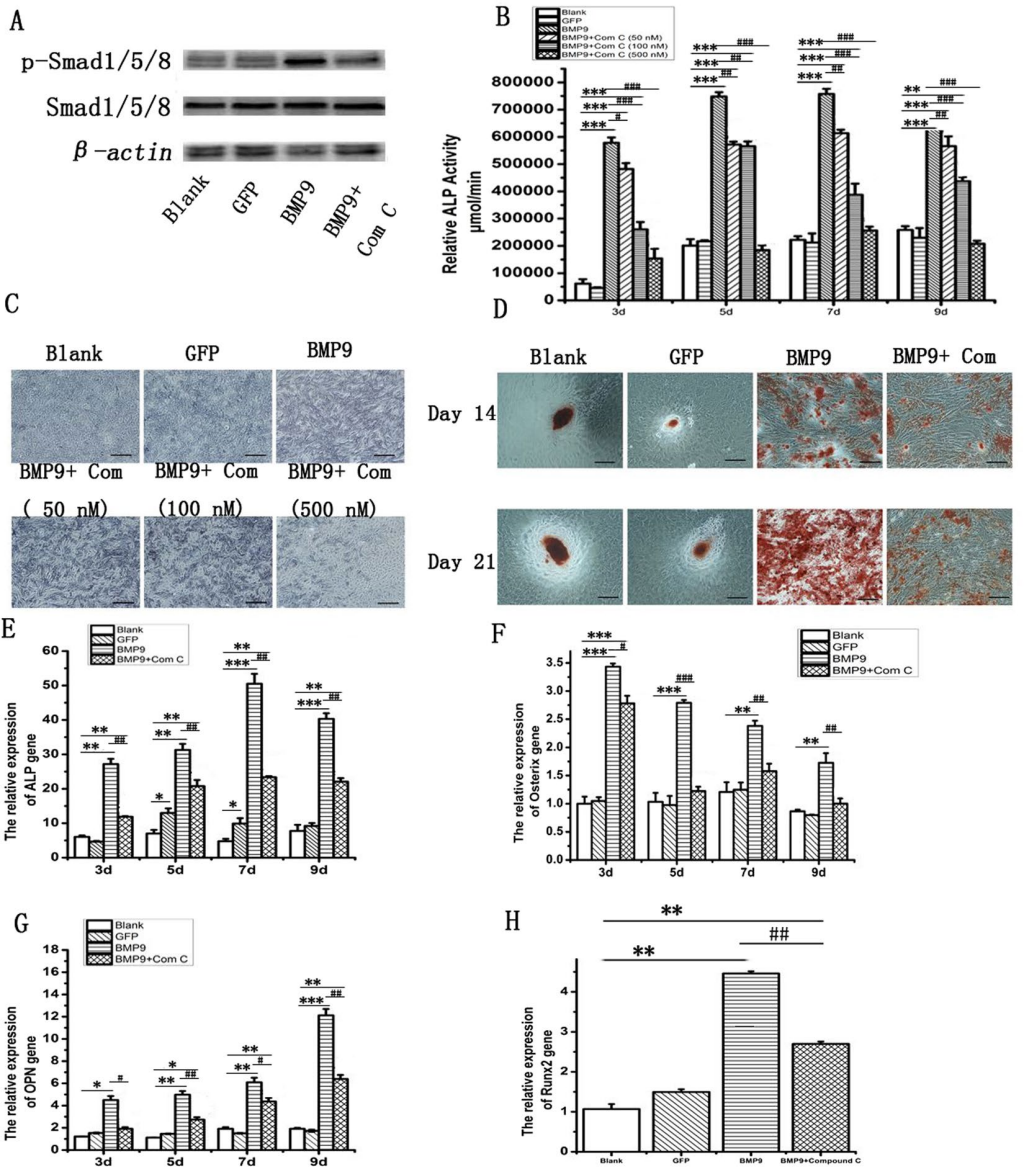
Activation of Smad1/5/8 signaling pathway is involved in BMP9-induced osteogenic differentiation of rDFCs. (**A**) Western Blot assay showed that BMP9 activated the Smad1/5/8 signaling pathway and increased the expression of phosphorylation Smad1/5/8. (**B**) ALP activity assay showed effect of Smad1/5/8 signaling pathway inhibition on rDFCs osteogenic differentiation by Compound C in a dose-dependent manner. (**C**) Effect of Smad1/5/8 signaling pathway inhibition on osteogenic differentiation of rDFCs in a concentration manner. ALP staining on day 7 was showed. Black bars: 100 μm. (**D**) Alizarin red staining was used to detect the calcium deposition on days 14 and 21. Black bars: 100 μm. (**E**,**F**,**G** and **H**) osteogenic markers detected by Real Time-*q*PCR on days 3, 5, 7, 9. (**E**) Alkaline phosphatase, ALP. (**F**) Osterix, (**G**) Osteopontin, OPN. (**H**) runt-related transcription factor 2, Runx2. *P < 0.05, compared with blank group, **P < 0.01, ***P < 0.001 compared with blank group, ^#^P < 0.05, ^##^P < 0.01, ^###^P < 0.001, compared with BMP9 group.

BMP proteins could act through Smad dependent or Smad independent manner. To investigate the roles of Smad pathway played in BMP9 induced rDFCs osteogenic differentiation, we first analyzed the Smad1/5/8 protein phosphorylation in those cell population. The results showed that Smad1/5/8 protein was induced in all groups and level of phosphorylated Smad1/5/8 protein (p-Smad1/5/8) was only significantly increased in Ad-BMP9-transfected rDFCs (Fig. 5A and Supplementary Fig. S1). We also applied the Smad1/5/8 pathway inhibitor (Compound C, Com C) into the cells culture.ALP activity, ALP staining, and Alizarin red staining results of showed that the osteogenic differentiation was attenuated by Com C in a dose-dependent manner (Fig. 5B,C and D). At the concentration of 500 nmol/L, the inhibitor achieved the maximal osteogenic inhibition effects. Meanwhile, we have verified the expression of osteogenic markers. The Real Time-*q*PCR results showed that the mRNA level of ALP, Osterix, and OPN were remarkably reduced by adding Smad1/5/8 pathway inhibitor (Fig. 5E,F,G and H). These findings indicated that Smad1/5/8 signaling pathway might be involved in BMP9-induce osteogenesis of rDFCs.

## Discussion

The three key elements of tissue engineering study are cells, growth factors and scaffolds. In this report, we demonstrated that rDFCs could thrive on the CHA scaffold and BMP9 could strongly promote the osteogenesis of rDFCs. Based on these findings, we architected a novel implant composed of Ad-BMP9-transfected rDFCs and CHA. This novel implant has been tested *in vivo* on a rat alveolar bone defect model. The results were very promising by showing the significant bone regeneration in the defected area. Our data also suggested that the Smad1/5/8 signaling pathway was involved in BMP9 promoted rDFCs osteogenic differentiation.

As one of the multipotent cells, rDFCs derive from ecto-mesenchymal tissue have the capacity to differentiate into osteoblasts and the potential for alveolar bone regeneration^38–40^. Our results demonstrated that rDFCs treated with Ad-BMP9 had strong ALP activity and mineralize deposition. Furthermore, Real Time-*q*PCR analysis of osteogenic marker genes including: ALP, Osterix, OPN, was significantly induced by BMP9 *in vitro*. These results indicated that BMP9 could promote osteogenic differentiation of rDFCs.

CHA scaffolds could serve as a cell carrier to repair the bone defects in previous studies and clinical cases^31, 41^. Due to the excellent shape and mechanical properties^41–43^, in this study, SEM data showed rDFCs could adhere to and then proliferated on the CHA. And the expression levels of OPN, ALP and Osterix were enhanced by CHA. This result indicated that CHA could also facilitate the osteogenic differentiation of rDFCs *in vitro* ^22, 24, 44, 45^. The diameter of CHA pores was ranging from 100 to 600 μm similar to the structure of cancellous, which was beneficial for cell-growth and vascularization in the formation stage of new bone^44, 46, 47^. The formation of a little new alveolar bone, due to the osteoblasts derived from the adjacent bone marrow cavities^48^, was found in CHA treatment group. This indicated that it did not achieve enough bone formation to repair alveolar bone defect in clinical by CHA scaffolds only. The combined CHA and rDFCs improved bone regeneration and mineralization compared to CHA alone according to the results of histological analysis and Micro CT study. And this effect might be related to the implanted rDFCs. The osteogenic activity of rDFCs might contribute to stimulate the resident osteoblasts and promote more new bone formation. However, the new bone was still limited to meet the alveolar bone defect.

To enhance more new bone formation, Ad-BMP9-transfected rDFCs combining with CHA were used. Some researchers reported that BMP9 had more potent in inducing osteogenesis of stem cells compared to other BMP family members^16, 46^. Recently, some successful researches *in vitro* and vivo suggested that the application of BMP9 could prove to be a useful approach to promote bone regeneration^11, 17, 37, 47^. In our study, immature fibrous-like bone with macrophagocytes and plenty of blood vessels were found in rDFCs/CHA group and BMP9-rDFCs/CHA group in immunodeficient mice. It cleared that the application of rDFCs could prove to be a useful approach to promote new bone regeneration. And our study also determined that whether the new combination therapy of BMP9-transfected rDFCs and CHA is possible for periodontal alveolar bone tissue engineering and for future clinical use. Histological studies in this study showed that the combination of BMP9-transfected rDFCs and CHA could significantly enhance the osteogenic differentiation of rDFCs and promote the osteogenic activity and alveolar bone regeneration. The Micro CT data verified this result and showed more new bone in BMP9/rDFCs/CHA group than other groups. BMP9 mediated the osteogenic activity of rDFCs and resident osteoblasts, and then promoted new bone formation. And the novel compound implants of Ad-BMP9-transfected rDFCs and CHA had the best effect in repairing the alveolar bone defect of rat. The tissue-engineered technique combined with BMP9, rDFCs and CHA achieved the best effects in repairing the alveolar bone defect of rat. However, there are many problems before clinical use, such as the security of this new bio-implant, the long-term effectiveness and the evaluation of this method.

BMP receptors and phosphorylated of Smad1/5/8 proteins are essential signaling mediators for BMP signaling pathway^19, 45, 49, 50^. The binding of BMP type I and type II receptors could introduce the phosphorylation of Smad1/5/8.Then, the phosphorylated Smad1/5/8 forms a heterodimeric complex with Smad4, which activates the transcription of target genes in the nucleus^45^. Compound C, a BMPs inhibitor, can block the BMP type I receptors (ALK1, ALK2, and ALK6) disrupting the phosphorylation of Smad1/5/8, without affecting MAPK activation^24^. In this study, our results revealed that the phosphorylated Smad1/5/8 proteins were significantly increased in BMP9 transfected rDFCs. It suggested that BMP9 could act through Smad1/5/8 signaling pathway in rDFCs osteogenesis. This hypothesis was further emphasized by finding that Compound C inhibited the osteogenic differentiation of BMP9-induced rDFCs. Thus, we suggested the activation of Smad1/5/8 signaling is required in BMP9-induced osteogenesis of rDFCs.

In conclusion, we have assembled a new type of implant with Ad-BMP9-transfected rDFCs and CHA. The BMP9 could boost the rDFCs osteogenic differentiation on the bio-degradable CHA scaffold through Smad signaling pathway. With this novel implants, the tissue-engineered technique combined with Ad-BMP9-transfected rDFCs and CHA achieved the best effects in new alveolar bone formation. These findings might lead to a novel approach in treating periodontitis related alveolar bone defects.

## Method

The consent procedure and experimental protocol were provided by laboratory of oral biomedical engineering of higher education of Chongqing Medical University (License: SCXK[YU]2012–0001). The experiments in the study were approved by the Ethics Committee of Chongqing Medical University. The procedures in the present study were in accordance with the National Institute of Health guidelines on the ethical use of animals^51^.

### Cells culture

Primary rDFCs were prepared and cultured as previously described^11^. Briefly, dental follicles were removed carefully from dental germs of neonatal rats’ mandibles. These cells were maintained in complete medium (DEME/F12, 10%FBS, 100 μg/mL streptomycin and 100 U/mL penicillin) and incubated at 37 °C in a humidified environment of 5% CO_2_. At the 3^rd^ passage, cells were re-suspended for further experiments. All animals were purchased from the Experimental Animal Center of Chongqing Medical University (License No: SCXK [Yu] 20012–0001).

### Ad-BMP9 infects rDFCs

80% confluence cells (passage 3) were digested by 0.25% trypsin, re-suspended and re-cultured in a humidified environment of 5% CO_2_ for 3 hours. Then, transfection was done with Ad-BMP9 at an appropriate MOI (multiplicity of infection, MOI, efficiency about 70%) followed by refreshing the basal medium in 4 hours. Adenovirus only expressing green fluorescence (Ad-GFP) was used as negative control. In blank experiment, polybrene (1 μM/mL) was only added^52, 53^. Cells were observed after 24 or 48 hours culture under the fluorescent microscope for quality control. Ad-BMP9 and Ad-GFP were provided by Dr. TC He (University of Chicago Medical Centre, USA).

### Detection of ALP activity

ALP activity was detected by chemiluminescence assay (alkaline phosphatase activity kit, Beyotime, China) and histochemistry staining (ALP staining Beyotime, China) as previously described^11^. Each assay condition was performed in triplicate and the results were determined by three independent experiments.

### Calcium deposition detection

Calcium deposition was stained with Alizarin Red S (Solarbio, Beijing, China), as previously described^11^. The cells were washed by PBS twice and incubated in 4% paraformaldehyde at room temperature for 30 minutes. After washed by double distilled water, the fixed cells were stained with 2% Alizarin Red Sat 37 °C for 20 minutes, followed by extensive washing by double distilled water. The staining results were recorded by an inverted microscope.

### Real Time-*q*PCR

The total mRNA was extracted by using RNA Extraction Kit (TaKaRa, Japan) on days 3, 5, 7, 9. cDNA was prepared by PrimeScript^TM^ RT reagent Kit (TaKaRa, Japan) according to the manufacturer’s instructions. Real Time-*q*PCR was employed to quantify the osteogenic gene expression (runt-related transcription factor 2, Runx2, alkaline phosphatase, ALP, osteopontin, OPN, Osterix) by using the SYBR green detection method as previously described^54^. All samples were run in triplicate and normalized by the expression of β-actin^55^. And the primers were synthesized by Genscript (Nanjing, China). The detailed primer information was as followed:

ALP, F: 5′-CCTGCAGGATCGGAACGTCAATTA-3′, R: 5′-TGAGTTGGTAAGG-CAGGGTCC-3′. OPN, F: 5′-CCAGCCAAGGACCAACTACA-3′, R: 5′-AGTGTT-TGCTGTAATGCGCC-3′. Runx2, F: 5′-CCGAGACCAACCGAGTCATTTA-3′, R: 5′-CCGAGACCAACCGAGTCATTTA-3′, Osterix, F: 5′-GCCAGTAATCTTCGTG-CCAG-3′, R: 5′-TAGTGAGCTTCTTCCTGGGGA-3′. β**-**actin, F: 5′-CCCGCGAG- TACAACCTTCTTG-3′,R: 5′-GTCATCCATGGCGAACTGGTG-3′.

### Observation of CHA by SEM

80% confluence cells were lifted in 0.25% tryptin,and re-suspended at a density of 2 × 10^6^ cells/ml onto CHA scaffolds (coralline hydroxyapatite, CHA, TBGC, China), which has been soaked in DMED medium containing 10% FBS for 2 hours. The cell-CHA combination was then cultured in a humidified environment with 5% CO_2_ at 37 °C. The medium was refreshed in every 3 days. The compound morphology of rDFCs and CHA (3 samples every day) was observed by SEM days 1 to days 7. At defined time points, the samples were rinsed twice in PBS and fixed in 2% glutaraldehyde for 2 hours before further preparation for SEM observation. Then, the scaffolds were washed by 0.1 mol/L solium-cacodylate for 30 minutes, dehydrated in a series of ethyl alcohol (50–100%) before being dried for SEM.

### Animal experiments

In order to investigate the osteogenesis of the new implantation (the compound of BMP9, rDFCs and CHA) without any stimulation, the ectopic bone formation experiments were done as follow: Total five immunodeficient mice were used in the ectopic implantation experiments. The transplants were prepared by culturing Ad-BMP9 transfected rDFCs on CHA for 7 days as previously described^56^. The immunodeficient mouse was anaesthetized by intraperitoneal injection of 1% pentobarbital (Nembutal 3.5 mg/100 g body weight). Four skin incisions about 1 cm in length were made on the dorsal surface of each mouse and implants (5samples each group) were placed into the pockets formed by blunt dissection. Then incisions were later closed by interrupted sutures. All mice were sacrificed after 6 weeks. The samples were immediately fixed in 4% paraformaldehyde (PH 7.4) for 48 hours at 4 °C followed by decalcification (15% EDTA -2Na solution, PH 7.4) and gradual dehydration before being embedded in paraffin wax. 5 μm thick sections were made and then stained with hematoxylin and eosin.

In order to test whether the novel implantation (the compound of BMP9, rDFCs and CHA) would support bone formation to restore alveolar bone defect in an acute traumatic inflammatory environment, the alveolar bone defect animal experiments were done as follow: Twenty SD rats were randomly divided into four groups in the alveolar bone regeneration experiments. The blank group received no implants. The CHA group was given CHA scaffolds only. The rDFCs/CHA group got 7-day-co-cultured rDFCs-CHA scaffolds. And the BMP9-rDFCs/CHA group acquired 7-day-co-cultured Ad-BMP9-transfected rDFCs-CHA scaffolds. For each experiment, rats were anaesthetized by intraperitoneal injection of 1% pentobarbital (Nembutal 3.5 mg/100 g body weight). The periapical alveolar bone of first and second molars was exposed by surgical means and an osseous defect (5 * 2 * 2 mm^3^) was created by round dental bur as previously described^54, 56, 57^. The defected area was then filled up with or without implants accordingly and covered by a collagen membrane (7 * 5 mm^2^). After that, the wound was closed with interrupted sutures. All animals were sacrificed 6 weeks after surgery. The alveolar bones were extracted and fixed in 4% paraformaldehyde (PH 7.4) for 48 hours at room temperature before Micro CT analysis or hematoxylin and eosin staining. To calculate the percentage of new bone formation over total defect for each group, at least 20 random high-power field of cross sections per group were evaluated by a picture-analysis system (the Image-Pro Plus software).The Micro-CT settings were as follows: pixel matrix, 2048 × 2048; Slice thickness, 10 µm; FOV/Diameter, 21.5 mm; Energy/intensity, 70 KVp, 114 µA, 8 W. 3D images were reconstructed by µCT-6.1 software (Scanco Vival CT 40, Switzerland)^57, 58^. Briefly, the threshold (205 to 500, represent gray value of new mineralization tissue) of gray value was selected. Another threshold (500 to 550) of gray value was regarded as old alveolar bone tissues. The values above 550 and below 205 were separately regarded as scaffold materials and void or soft tissues. Where scaffolds were was in red in 3D reconstruction images. The volume of interest (VOI) was a 5 mm^3^ cubic volume selected at the central defects in the threshold (205 to 500) from 3D images (n = 5), and the VOI was used to access the new bone formation.

### Regulation of osteogenic differentiation of BMP9 transfected rDFCs by Smad1/5/8 signal pathway

Cells were collected and lysed as previous described^22^. Rabbit anti-rat Smad1/5/8 (1:500) (Santa Cruz, USA), Rabbit anti-rat p-Smad1/5/8 (1:200) (CST, USA), Anti-β**-**actin antibody (1:1000) (Cell Signaling Technology, USA), were incubated with PVDF membrane respectively overnight at 4 °C, followed by the incubation with secondary antibody Goat anti-rabbit (1:3000) (Sino Biological, Beyotime, Shanghai, China). The ECL Chemiluminescent Substrate Kit was utilized to detect the target proteins. All experiments were carried out in triplicate.

Re-suspended cells were incubated and tansfection was done with Ad-BMP9 or Ad-GFP at an appropriate MOI (transfection efficiency about 70%) followed by supplement with different concentration of Compound C (0 nM, 50 nM, 100 nM, 500 nM). ALP activity was assayed by chemiluminescence assay (alkaline phosphatase activity kit, Beyotime, China) and histochemistry staining assay (ALP staining Beyotime, China) as previously described on day 7. And the detection of calcium deposition was done by Alizarin Red S. The osteogenic genes were detected by Real Time-*q*PCR. Each assay condition was performed in triplicate and the results were determined by three independent experiments.

### Statistical analysis

All experiments in our present study were repeated three times. The values in this study were shown as means ± standard deviation (S.D.). Differences were analyzed by student’s t-test or one-way analysis of variance (ANOVA) in SPSS (SPSS 19.0). *P* < 0.05 was considered as statistically significant.

### Ethical statement

The procedures in the present study were in accordance with the National Institute of Health guidelines on the ethical use of animals. The experiments in the study were approved by the Ethics Committee of Chongqing Medical University and the experimenter had also got the license of animal experiments (License No: CQLA-2014–0678). All animals were purchased from the Experimental Animal Center of Chongqing Medical University (License No: SCXK [Yu] 20012–0001).

## Electronic supplementary material

Supplementary Information

## References

[1] Jeftha A, Holmes H (2013). Periodontitis and cardiovascular disease. SADJ: journal of the South African Dental Association = tydskrif van die Suid-Afrikaanse Tandheelkundige Vereniging.

[2] Lim JC, Mitchell CH (2012). Inflammation, Pain, and Pressure-Purinergic Signaling in Oral Tissues. Journal of Dental Research.

[3] Bartold PM, Mcculloch CAG, Narayanan AS, Pitaru S (2000). Tissue engineering: a new paradigm for periodontal regeneration based on molecular and cell biology. Periodontology.

[4] Bono A, Brunotto M (2010). Amoxicillin/metronidazole or scaling and root planing in the treatment of chronic periodontitis. Acta Odontológica Latinoamericana Aol.

[5] Kodama T (2013). Guided tissue regeneration using a collagen barrier and bone swaging technique in noncontained infrabony defects. International Journal of Periodontics & Restorative Dentistry.

[6] Wood RA, Mealey BL (2012). Histologic comparison of healing after tooth extraction with ridge preservation using mineralized versus demineralized freeze-dried bone allograft. Journal of Periodontology.

[7] Jiawen S (2014). Osteogenic differentiation of human amniotic epithelial cells and its application in alveolar defect restoration. Stem Cells Translational Medicine.

[8] Langer R (2000). Tissue engineering. Science.

[9] Polimeni G, Xiropaidis AV, Wikesjö UME (2006). Biology and principles of periodontal wound healing/regeneration. Periodontology.

[10] Yao S, Pan F, Prpic V, Wise GE (2008). Differentiation of stem cells in the dental follicle. Journal of Dental Research.

[11] Li C (2012). Bone Morphogenetic Protein-9 Induces Osteogenic Differentiation of Rat Dental Follicle Stem Cells in P38 and ERK1/2 MAPK Dependent Manner. International Journal of Medical Sciences.

[12] Morsczeck C (2005). Isolation of precursor cells (PCs) from human dental follicle of wisdom teeth. Matrix Biology.

[13] Völlner F, Ernst W, Driemel O, Morsczeck C (2009). A two-step strategy for neuronal differentiation *in vitro* of human dental follicle cells. Differentiation.

[14] Rad MR (2015). The role of dentin matrix protein 1 (DMP1) in regulation of osteogenic differentiation of rat dental follicle stem cells (DFSCs). Archives of Oral Biology.

[15] Zuo-lin (2008). Osteogenic-related gene expression profiles of human dental follicle cells induced by dexamethasone. Acta Pharmacologica Sinica.

[16] Kang Q (2004). Characterization of the distinct orthotopic bone-forming activity of 14 BMPs using recombinant adenovirus-mediated gene delivery. Gene Therapy.

[17] Kimelman BN (2012). Gene therapy approaches to regenerating bone. Advanced Drug Delivery Reviews.

[18] Luu HH (2007). Distinct roles of bone morphogenetic proteins in osteogenic differentiation of mesenchymal stem cells †. Journal of Orthopaedic Research Official Publication of the Orthopaedic Research Society.

[19] Li R (2016). The Prodomain-Containing BMP9 Produced from a Stable Line Effectively Regulates the Differentiation of Mesenchymal Stem Cells. International Journal of Medical Sciences.

[20] Xu DJ (2012). Smads, p38 and ERK1/2 are involved in BMP9-induced osteogenic differentiation of C3H10T1/2 mesenchymal stem cells. Bmb Reports.

[21] Dumont RJ (2002). *Ex vivo* bone morphogenetic protein-9 gene therapy using human mesenchymal stem cells induces spinal fusion in rodents. Neurosurgery.

[22] Shui W (2014). Characterization of scaffold carriers for BMP9-transduced osteoblastic progenitor cells in bone regeneration. Journal of Biomedical Materials Research Part A.

[23] Wang J (2017). NEL-Like Molecule-1 (Nell1) Is Regulated by Bone Morphogenetic Protein 9 (BMP9) and Potentiates BMP9-Induced Osteogenic Differentiation at the Expense of Adipogenesis in Mesenchymal Stem Cells. Cellular Physiology & Biochemistry International Journal of Experimental Cellular Physiology Biochemistry & Pharmacology.

[24] Ye J (2016). A thermoresponsive polydiolcitrate-gelatin scaffold and delivery system mediates effective bone formation from BMP9-transduced mesenchymal stem cells. Biomedical Materials.

[25] Chang H, Brown CW, Matzuk MM (2003). Genetic analysis of the mammalian transforming growth factor-beta superfamily. Endocrine Reviews.

[26] López-Coviella I, Berse B, Krauss R, Thies RS, Blusztajn JK (2000). Induction and maintenance of the neuronal cholinergic phenotype in the central nervous system by BMP-9. Science.

[27] Anai M (2011). Activation of Bmp2-Smad1 signal and its regulation by coordinated alteration of H3K27 trimethylation in Ras-induced senescence. IEEE Transactions on Computer-Aided Design of Integrated Circuits and Systems.

[28] Valentinopran A, Wozney J, Csimma C, Lilly L, Riedel GE (2002). Clinical evaluation of recombinant human bone morphogenetic protein-2. Clinical Orthopaedics & Related Research.

[29] Luther G (2011). BMP-9 induced osteogenic differentiation of mesenchymal stem cells: molecular mechanism and therapeutic potential. Current Gene Therapy.

[30] Ning Y (2009). The research of degradability of a novel biodegradable coralline hydroxyapatite after implanted into rabbit. Journal of Biomedical Materials Research Part A.

[31] Georgiannos D, Lampridis V, Bisbinas I (2015). Phenolization and coralline hydroxyapatite grafting following meticulous curettage for the treatment of enchondroma of the hand. A case series of 82 patients with 5-year follow-up. Hand.

[32] Ripamonti U (1991). The morphogenesis of bone in replicas of porous hydroxyapatite obtained from conversion of calcium carbonate exoskeletons of coral. Journal of Bone & Joint Surgery.

[33] Irwin RB, Bernhard M, Biddinger A (2001). Coralline hydroxyapatite as bone substitute in orthopedic oncology. American Journal of Orthopedics.

[34] Mygind T (2007). Mesenchymal stem cell ingrowth and differentiation on coralline hydroxyapatite scaffolds. Biomaterials.

[35] Ye G (2013). Bone morphogenetic protein-9 induces PDLSCs osteogenic differentiation through the ERK and p38 signal pathways. International Journal of Medical Sciences.

[36] Liu Y (2014). All-trans retinoic acid modulates bone morphogenic protein 9-induced osteogenesis and adipogenesis of preadipocytes through BMP/Smad and Wnt/β-catenin signaling pathways. International Journal of Biochemistry & Cell Biology.

[37] Xiang L, Liang C, Zhen-Yong K, Liang-Jun Y, Zhong-Liang D (2011). BMP9-induced osteogenetic differentiation and bone formation of muscle-derived stem cells. Biomed Research International.

[38] Takahashi K (2015). Applicability of human dental follicle cells to bone regeneration without dexamethasone: an *in vivo* pilot study. International Journal of Oral & Maxillofacial Surgery.

[39] Rad, M. R. Characteristics of Dental Follicle Stem Cells and Their Potential Application for Treatment of Craniofacial Defects (2014).

[40] Rezai-Rad M (2015). Evaluation of bone regeneration potential of dental follicle stem cells for treatment of craniofacial defects. Cytotherapy.

[41] Koëter S (2008). Coralline hydroxyapatite is a suitable bone graft substitute in an intra-articular goat defect model. Journal of Biomedical Materials Research Part B Applied Biomaterials.

[42] Shors EC (1999). Coralline bone graft substitutes. Orthopedic Clinics of North America.

[43] Nandi SK (2015). Converted marine coral hydroxyapatite implants with growth factors: *In vivo* bone regeneration. Materials Science & Engineering C.

[44] Zhang R (2015). BMP9-induced osteogenic differentiation is partially inhibited by miR-30a in the mesenchymal stem cell line C3H10T1/2. Journal of Molecular Histology.

[45] Lamplot JD (2013). BMP9 signaling in stem cell differentiation and osteogenesis. American Journal of Stem Cells.

[46] Fujioka-Kobayashi, M. *et al*. Osteogenic potential of rhBMP9 combined with a bovine-derived natural bone mineral scaffold compared to rhBMP2. *Clinical Oral Implants Research* (2016). 10.1111/clr.12804 26988608

[47] Wu N, Zhao Y, Yin Y, Zhang Y, Luo J (2010). Identification and analysis of type II TGF-β receptors in BMP-9-induced osteogenic differentiation of C3H10T1/2 mesenchymal stem cells. Acta Biochimica et Biophysica Sinica.

[48] Einhorn TA (1998). The Cell and Molecular Biology of Fracture Healing. Clinical Orthopaedics & Related Research.

[49] Hogan BL (1996). Bone morphogenetic proteins: multifunctional regulators of vertebrate development. Genes & Development.

[50] Yu PB (2007). Dorsomorphin inhibits BMP signals required for embryogenesis and iron metabolism. Nature Chemical Biology.

[51] The ministry of Science and Technology of the People’s Republic of China. Guidance for experimental animal care. 2006-09-30. PRC (2006).

[52] Kim, S.-Y., Lee, S.-J., Han, H.-K. & Lim, S.-J. Aminoclay as a highly effective cationic vehicle for enhancing adenovirus-mediated gene transfer through nanobiohybrid complex formation (2016). 10.1016/j.actbio.2016.11.045 27872011

[53] Han M (2015). Polybrene: Observations on cochlear hair cell necrosis and minimal lentiviral transduction of cochlear hair cells. Neuroscience Letters.

[54] Liang Y (2016). Endothelial progenitors enhanced the osteogenic capacities of mesenchymal stem cells *in vitro* and in a rat alveolar bone defect model. Archives of Oral Biology.

[55] Ling X (2012). The healing of critical-size calvarial bone defects in rat with rhPDGF-BB, BMSCs, and β-TCP scaffolds. Journal of Materials Science: Materials in Medicine.

[56] Yong XL (2015). *In vitro* and *In vivo* Evaluation of the Developed PLGA/HAp/Zein Scaffolds for Bone-Cartilage Interface Regeneration. Biomedical & Environmental Sciences.

[57] Xu L (2012). The healing of critical-size calvarial bone defects in rat with rhPDGF-BB, BMSCs, and β-TCP scaffolds. Journal of Materials Science Materials in Medicine.

[58] Komlev VS (2010). Biodegradation of porous calcium phosphate scaffolds in an ectopic bone formation model studied by X-ray computed microtomograph. European cells & materials.

