# Supplementary material for: The healing of alveolar bone defects with novel bio-implants composed of Ad-BMP9-transfected rDFCs and CHA scaffolds
Source: Sci Rep. 2017 Jul 25;7:6373. doi: 10.1038/s41598-017-06548-7 (PMC5527078; doi:10.1038/s41598-017-06548-7)

The healing of alveolar bone defects with novel bio-implants composed of Ad-BMP9-transfected rDFCs and CHA scaffolds

Li Nie[a b c 1], Xia Yang[a b c 1], Liang Duan[d], Enyi Huang[a b c], Pengfei, Zhou[a b c], Wenping Luo[a b c],Yan Zhang[a b c], Xingqi Zeng[a b c], Ye Qiu[a b c], Ting Cai[a b c], Conghua Li[a b c] *

[a]Stomatological Hospital of Chongqing Medical University, Chongqing, 401147, China

[b]Chongqing Key Laboratory of Oral Diseases and Biomedical Sciences, Chongqing Municipal Key, Chongqing, 401147, China

[c]Laboratory of Oral Biomedical Engineering of Higher Education，Chongqing, 401147, China

[d]Department of Laboratory Medicine, the Second Affiliated Hospital of Chongqing Medical University, Chongqing, 400010, China

[1] These authors contributed equally to this work

[*] Correspondence should be addressed to C.L. (email: liconghua1@163.com).

Address: Stomatological Hospital of Chongqing Medical University, Chongqing, 401147, China

Tel.:86-23-65714733,

Supp. Fig. S1. Full-length blots/gels are presented.

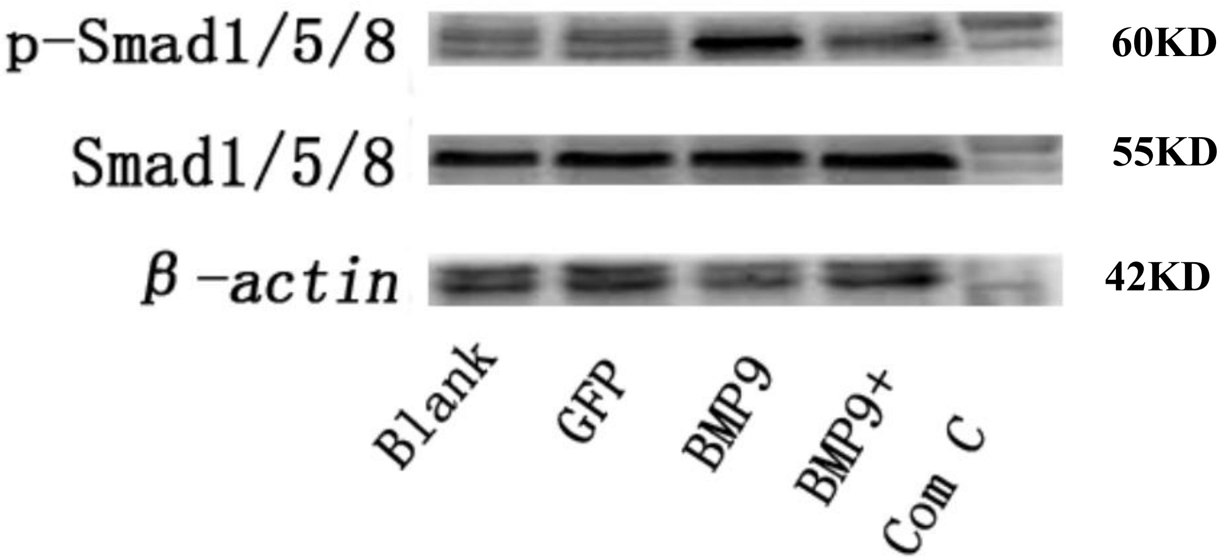

Supplement: Supplementary file 1 — Supplementary Information [file 41598_2017_6548_MOESM1_ESM.pdf]
